# Naive Bayes Bearing Fault Diagnosis Based on Enhanced Independence of Data

**DOI:** 10.3390/s18020463

**Published:** 2018-02-05

**Authors:** Nannan Zhang, Lifeng Wu, Jing Yang, Yong Guan

**Affiliations:** 1 College of Information Engineering, Capital Normal University, Beijing 100048, China; zhangnn@cnu.edu.cn (N.Z.); yangjing@cnu.edu.cn (J.Y.); guanyong@cnu.edu.cn (Y.G.); 2 Beijing Key Laboratory of Electronic System Reliability Technology, Capital Normal University, Beijing 100048, China; 3 Beijing Key Laboratory of Light Industrial Robot and Safety Verification, Capital Normal University, Beijing 100048, China; 4 Beijing Advanced Innovation Center for Imaging Technology, Capital Normal University, Beijing 100048, China

**Keywords:** Naive Bayes, decision tree, support vector machines, fault diagnosis

## Abstract

The bearing is the key component of rotating machinery, and its performance directly determines the reliability and safety of the system. Data-based bearing fault diagnosis has become a research hotspot. Naive Bayes (NB), which is based on independent presumption, is widely used in fault diagnosis. However, the bearing data are not completely independent, which reduces the performance of NB algorithms. In order to solve this problem, we propose a NB bearing fault diagnosis method based on enhanced independence of data. The method deals with data vector from two aspects: the attribute feature and the sample dimension. After processing, the classification limitation of NB is reduced by the independence hypothesis. First, we extract the statistical characteristics of the original signal of the bearings effectively. Then, the Decision Tree algorithm is used to select the important features of the time domain signal, and the low correlation features is selected. Next, the Selective Support Vector Machine (SSVM) is used to prune the dimension data and remove redundant vectors. Finally, we use NB to diagnose the fault with the low correlation data. The experimental results show that the independent enhancement of data is effective for bearing fault diagnosis.

## 1. Introduction

The rolling bearing is the main component of rotating machinery. It carries the entire rotating machinery and equipment operation, and a small fault may have a significant impact on the operation of the entire device. Most of the problems with rotating machines are caused by bearing failure [1]. Therefore, the bearing fault diagnosis is of great significance. After the fault diagnosis of rotating machinery, the machine can be repaired and handled in time, so as to avoid the catastrophic effect caused by mechanical failure [2]. The related contents and techniques of fault diagnosis are introduced in the literature [3,4,5,6]. Before the machine failure, the maintenance and treatment of the machine can prevent the probability of failure and reduce the maintenance costs of the machine, as well as avoid casualties caused by equipment failure.

The vibration analysis is the main tool for the diagnosis of rotating machinery [7], and vibration signals analysis has been widely used in the field of fault diagnosis. In this field, the vibration spectrum analysis technique has successful identified the faults [8,9,10,11]. Through the analysis of vibration signals, the state of rotating machinery can be reflected. Sensors can be used to collect vibration signals of operating machinery, which contains rich information about the working state of machinery [12]. The mechanical health state is determined by analyzing the collected vibration signals. However, the collected vibration signals are chaotic and irregular. Therefore, it is necessary to extract the most representative, reliable and effective features from the acquired vibration signals.

The time domain signal feature of statistical analysis can be used to detect faults, which is mainly to extract feature of data. However, it can only reflect whether rotating machinery and its state are normal, and it can give diagnostic messages but not diagnose the fault, so further fault diagnosis is needed. Nowadays, with the successful application of machine learning methods in various fields, more and more machine learning methods are used in mechanical fault diagnosis. Neural networks, as a typical method of machine learning, has been applied to the field of fault diagnosis. As the most popular classifier, Support Vector Machines (SVM) has achieved some success in the field of fault diagnosis. SVM is a powerful tool for classification, and it also plays a significant role in machine fault diagnosis [13]. Samanta [14] proposed time-domain characteristics of the rotating machine that can be used as the input of artificial neural network (ANN) to verify the effective application in bearing fault diagnosis. Jack et al. [15] put forward SVM for bearing fault diagnosis. Yang et al. [16] proposed that vibration signals can be decomposed to stationary intrinsic mode functions (IMFs), and the input of ANN is the energy features extracted from IMF so as to identify the rolling bearing failure. Al-Raheem et al. [17] proposed a new technique that used genetic algorithm to optimize the application of Laplace wavelet shape and classifier parameters of ANN for bearing faults. In addition, Shuang et al. [18] proposed a fault pattern recognition method based on the principal component analysis (PCA) and SVM. However, the extracted multi-dimensional feature vector contains a large amount of information, with high data redundancy, which results in higher computational costs. Therefore, the high-dimensional characteristics need to be processed. Wu et al. [19] used the Manifold Learning algorithm to reduce the dimension of the high-dimensional features and then the processed are used as the input of wavelet neural network for bearing fault diagnosis. Sugumaran et al. [20] applied Decision Tree to selecting feature, and then carried on the bearing fault diagnosis with the kernel neighborhood fractional multiple support vector machine (MSVM). In another article [21], first, the time domain statistical feature and histogram feature was extracted from time domain signals, then the main feature was selected by the Decision Tree, last SVM and Proximal Support Vector was used for fault diagnostics of roller bearing. In a recent study, Ran et al. [22] proposed a neural network-based method to directly identify the original time series sensor data without feature selection and signal processing for bearing fault diagnosis. In his other article [23], the network is a combination of linear and nonlinear method, and also uses the depth network classifier of the original time series sensor data to diagnose faults.

ANN and SVM have an extensive application in fault diagnosis. However, there are some limitations. For example, the fitting problem and the local extremum can lead to slow operation speed and inaccurancy in ANN training results, respectively [24]. Moreover, SVM has a problem with the speed of testing and training. There are some limitations on multi-class, nonlinear and parameters problem. The training of ANN and SVM is complex, and the cost on training space is high. NB is used not only a small mount of training data, but also its simple structure, fast calculation speed and high accuracy [25,26]. Due to the reliable theoretical basis, comprehensive prior knowledge and the assumption of independent conditions among attributes, NB successfully applied in machine fault diagnosis. Hemantha et al. used the Bayes classifier to diagnose the bearing fault, and verified that NB on fault diagnosis has a good performance [27]. Girish et al. successfully applied NB classifier to the welded joints fault diagnosis [28]. However, the independence assumption of vibration signal of bearing fault is difficult to be realized in actual situations, which limits the algorithm. Therefore, this paper mainly carries on the vector pruning from two aspects of the characteristic attributes and the data dimension. First, Decision Trees are mainly used to select the main feature attributes [29]. Then, the redundancy of dimension vectors is removed by the proposed selective support vector machine (SSVM). In this way, the redundant data is processed from two aspects, and the limitation of the independence hypothesis on the NB is reduced. Finally, fault diagnosis model is established.

In this paper, NB, which is based on data independence improvement in fault diagnosis, is proposed. The remainder of paper is organized as follows: in Section 2, there is a brief introduction of the NB model. The fault diagnosis based on improved data independence is given by Section 3. In Section 4, the fault diagnosis based on improved data independence is applied to roller bearing diagnosis. Section 5 draws the conclusion of this paper.

## 2. NB Model

**Figure 1:**
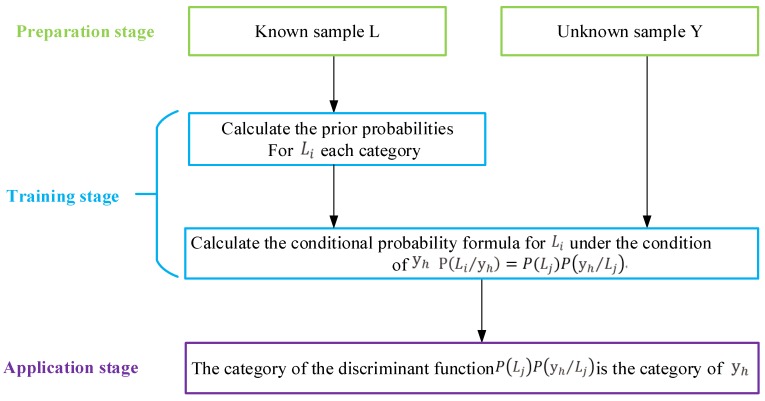
Naive Bayes training model.

NB is a supervised learning classification method based on probability. NB has received much attention due to its simple classification model and excellent classification performance. The training model is shown in Figure 1.

(a)   Preparatory stage

Suppose there are *m* categories and categories $L=\{L_{1},L_{2},\cdots ,L_{m}\}$. Each sample has *n* attributes $At=\{At_{1},At_{2},\cdots ,At_{n}\}$, and each attribute set has *d*-dimensional feature vector $X=\{X_{1},X_{2},\cdots ,X_{d}\}$.

(b)   Training stage

$P(L_{i})$ is the prior probability of each category, only related to the ratio of each category to the total category, that is,

(1) $$P\left(L_{i}\right)=\frac{n_{i}}{n},1\leq i\leq m,$$

where *n* is the number of known sample, and $n_{i}$ is the number of *i*-th categories.

Bayes is a classifier based on the maximum posterior probability. There is an unknown sample classes $Y=\{y_{1},y_{2},\cdots ,y_{z}\}$, and the idea is to calculate the probability of unknown samples in each category. Finally, if the probability of the unknown sample *Y* is maximum in class $L_{i}$, the unknown sample is classified into category $L_{i}$. NB is based on the Bayes theorem, and the NB classification method is shown below:

(2) $$P\left(L_{i}/y_{h}\right)>P\left(L_{j}/y_{h}\right),1\leq i\leq m,1\leq j\leq m,1\leq h\leq z,i\neq j.$$

According to Bayes’s theorem, the probability formula of $P(L_{i}/y_{h})$ can be obtained. The NB is a Bayes theorem based on the independence of the characteristic conditions, so $P(L_{i}/y_{h})$ can be defined as follows:

(3) $$P\left(L_{i}/y_{h}\right)=\frac{P\left(y_{h}/L_{i}\right)P\left(L_{i}\right)}{P(y_{h}),}$$

(4) $$P\left(y_{h}\right)=\sum_{i=1}^{m}P\left(y_{h}/L_{i}\right)P\left(L_{i}\right),$$

where $P(y_{h})$ is a constant, and it is only necessary to compute the formula $P\left(y_{h}/L_{i}\right)P\left(L_{i}\right)$ of Equation (3).

According to the NB classification method, the value of the discriminant function $P\left(y_{h}/L_{i}\right)P\left(L_{i}\right)$ in each class is calculated for the unknown sample, where $P(L_{i})$ is a priori probability of each category, as shown in Equation (1), and where $P(y_{h}/L_{i})$ is the probability of $y_{h}$ under the condition of $L_{i}$. The attribute $At_{gi}$ is continuous property and independent of each other. In general, the attribute variable obeys the Gaussian distribution $At_{gi}∼N\left(u_{gi},\delta_{gi}^{2}\right)$ [30]; then, $P(y_{h}/L_{i})$ is defined as follows:

(5) $$P\left(y_{h}/L_{i}\right)=\frac{1}{\sqrt{2\pi }\delta_{gi}}exp\left\{-\frac{{\left(y_{h}-u_{gi}\right)}^{2}}{2\delta_{gi}^{2}}\right\},$$

where $u_{gi}$ and $\delta_{gi}^{2}$ are mean and variance of samples, respectively, and the formula is as follows:

(6) $$u_{gi}=\frac{\sum_{i=1}^{n_{i}}X_{ig}}{n_{i}},$$

(7) $$\delta_{gi}^{2}=\frac{\sum_{i=1}^{n_{i}}\left(X_{ig}-u_{gi}\right)}{n_{i}-1}.$$

From the above Equations (2) and (5)–(7), the posterior probability equation can be obtained:

(8) $$P\left(L_{i}/y_{h}\right)=P\left(L_{i}\right)\prod_{g=1}^{n}\frac{1}{\sqrt{2\pi }\delta_{gi}}exp\left\{-\frac{{\left(y_{h}-u_{gi}\right)}^{2}}{2\delta_{gi}^{2}}\right\}.$$

In the same way:

(9) $$P\left(L_{j}/y_{h}\right)=P\left(L_{j}\right)\prod_{g=1}^{n}\frac{1}{\sqrt{2\pi }\delta_{gj}}exp\left\{-\frac{{\left(y_{h}-u_{gj}\right)}^{2}}{2\delta_{gj}^{2}}\right\}.$$

(c)   Application stage  

According to the Equation (2), if $P\left(L_{i}\right)P\left(L_{i}/y_{h}\right)>P\left(L_{j}\right)P\left(L_{j}/y_{h}\right)$, the unknown sample is judged as class *i*; otherwise, it is judged as *j*.

## 3. NB Fault Diagnosis Model Based on Enhanced Independence of Data

### 3.1. Fault Diagnosis Model

**Figure 2:**
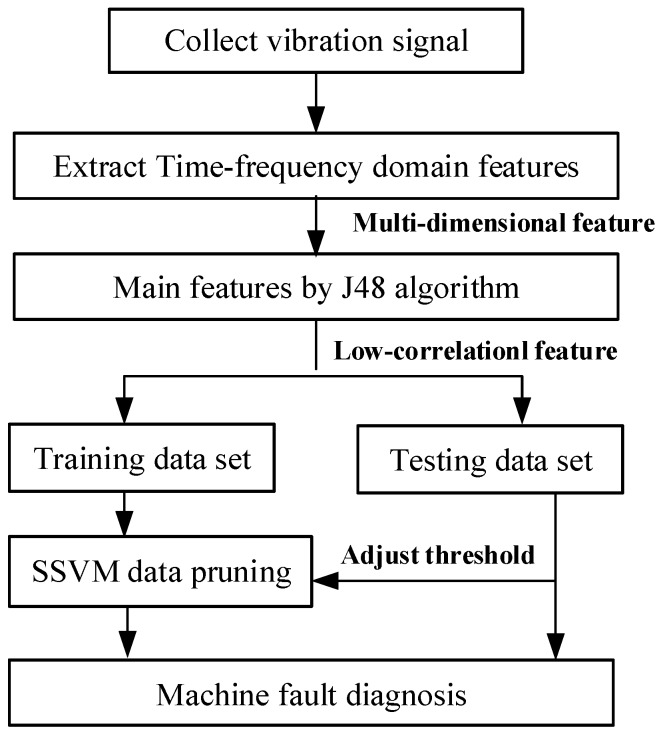
Fault diagnosis model based on the enhanced independence of data.

In order to improve the classification effect of NB, this paper enhances the independence between data from two aspects of attribute characteristics and data dimension. The proposed fault diagnosis model is shown in Figure 2. The fault diagnosis model includes three parts: signal acquisition, signal processing and fault diagnosis.

- Signal acquisition:   Acceleration sensor is used to obtain vibration signals of rolling bearings.
- Signal processing:   The original vibration signal of the rolling bearing obtained from the sensor contains a large amount of noise, so it is necessary to process the data to obtain valid data signals. Firstly, feature extraction is performed on the original signal acquired by using the time-domain signal method. Then, the Decision Tree is used to select the main feature attributes from the feature attributes. The data are processed from two directions of feature attribute and data dimension, so that the data with strong independence can be obtained, which is beneficial to the fault diagnosis of the bearing.
- Fault diagnosis:  After the data is processed, we obtain data with low redundancy. Thus, the impact of data independence assumption on NB model is reduced, and the fault diagnosis can be made effectively.

### 3.2. Feature Selection Using Decision Tree

The Decision Tree is a tree structure, which is mainly composed of nodes and branches, and the nodes contain leaf nodes and intermediate nodes. The intermediate nodes are used to represent a feature, and leaf nodes are used to represent a class label. The Decision Tree can be used for feature selection [29]. The attributes that appear in the Decision Tree nodes provide important information to promote classification. The J48 algorithm is mainly used to construct Decision Tree. Therefore, we construct a Decision Tree using J48 algorithm. Then, we find the characteristic attribute corresponding to the middle node of the decision tree, and remove the feature attribute that without important information. The following describes the J48 algorithm for feature extraction:

(a) The acquired data is used as the input of the algorithm, and the output is the node of the Decision Tree.
(b) The output Decision Tree nodes are divided into leaf nodes and intermediate nodes. The leaf node represents the classification, the intermediate node represents the decision attribute, and the branch represents the condition that the next decision data comes from the previous decision attribute.
(c) The Decision Tree is used to find feature attributes from top to bottom until all nodes become leaf nodes.
(d) Finding the criteria of decision attributes: the information gain of each feature is calculated and the maximum information gain is chosen as the intermediate node of the Decision Tree.

Information gain is used to determine how to select the most appropriate features from a number of attributes. Information gain is mainly determined by the information entropy. Information gain of attribute At for the data set is: entropy of all attribute information minus the entropy of split attributes. The At is a continuous attribute based on Gaussian distribution, so information entropy properties of At is defined as follows:

(10) $$Gain\left(At\right)=Info\left(L\right)-info_{At}\left(L\right),$$

(11) $$Info\left(L\right)=-\sum_{j=1}^{m}P\left(j/L\right)logP\left(j/L\right),$$

(12) $$Info_{At}\left(L_{j}\right)=\sum_{j=1}^{m}\frac{L_{j}}{L}\frac{Log2\pi e\delta_{ij}^{2}}{2}.$$

Gain(At) is the information gain of the attribute At, Info(L) is the undivided information entropy, and $info_{At}\left(L\right)$ is the information entropy At after splitting. The variance *x* is given by the Formula (6), and m is the number of classifications, and $L_{j}$ is a subset of data set *L*.

### 3.3. SSVM

SVM is a traditional classification method for two categories. In this paper, an optimal classification hyperplane is constructed in the sample set, and two classes of samples are separated from each other on the hyperplane. Generally, in the case of too much data, SVM can not completely classify the two kinds of data into both sides of the hyperplane. Thus, we propose an SSVM algorithm to remove the spatial redundancy problem of the vector.

**Figure 3:**
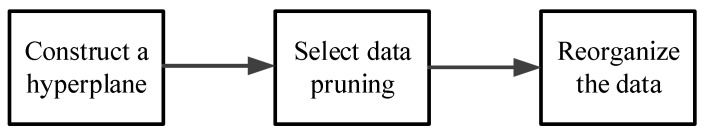
Selective Support Vector Machine data processing flow chart.

SSVM data processing is divided into several steps, as shown in Figure 3.

Step 1: Constructing the optimal hyperplane of data.  

**Figure 4:**
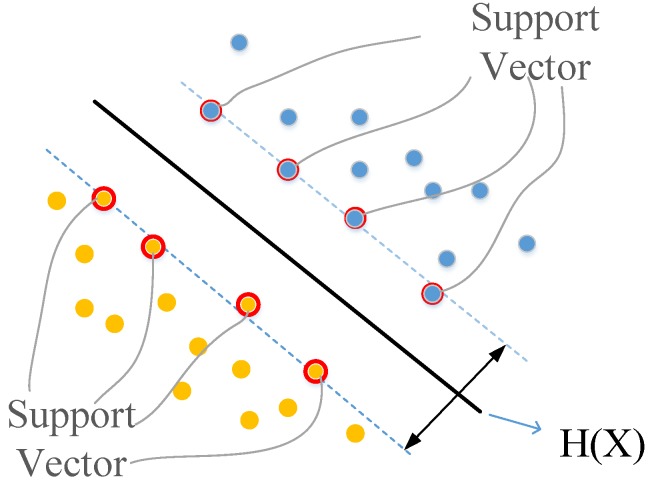
Two categories of Support Vector Machine.

In most cases, SVM is targeted at two types of problems [31]. The data set (X,Y) is divided into training set and test set. The training set is $\left(X_{1},Y_{1}\right),\left(X_{2},Y_{2}\right),\cdots ,\left(X_{n},Y_{n}\right)$. if $X_{i}$ is the first class, $Y_{i}=1$. if $X_{i}$ is second class, $Y_{i}=-1$. As shown in Figure 4, hyperplane H(X) separates the two-class data on both sides.

The hyperplane H (X) equation is given as in Equation (13) [32]:

(13) $$w^{T}K\left(X\right)+b=0.$$

The function K(X) is a kernel function, which maps the low-dimensional space to the high-dimensional space, and avoids the fact that the data cannot be separated in the low-dimensional space, where w is a vector, b is constant, and their values can be obtained by the optimization of the following Equation [31]:

(14) $$min:\frac{1}{2}∥w∥+C\sum_{i=1}^{n}\xi_{i},∥w∥=w^{T}w,$$

(15) $$s.t.\;y_{i}\left(w^{T}K\left(X_{i}\right)+b\right)\geq 1-\xi_{i},\xi_{i}\geq 0.$$

Parameter *C* is mainly used to adjust training error. $\xi_{i}$ is a slack variable [33]. After the solution of the parameter, the optimal hyperplane H(X) is obtained [31]:

(16) $$H\left(X\right)=sgn(\sum_{i\varepsilon SV}y_{i}a_{i}K\left(x_{i},x\right)+b),$$

where *S* is the support vector for the DataSet (X,Y), where sgn is a symbolic function that mainly returns the positive and negative of the parameter. $K(x_{i},x)$ is a kernel function, and there are many kinds of kernel functions. The Gaussian kernel function is better in the application, so the Gaussian kernel function is used in this paper:

(17) $$K\left(x,y\right)=exp\left(\frac{∥x-y∥}{2\sigma^{2}}\right).$$

Step 2: Using the constructed hyperplane to select the data and remove the redundancy.  

Firstly, a suitable threshold is selected, and the hyperplane K(X) is used to test the data. When the test result does not reach the threshold, this data is chosen to be pruned.

Then, find the hyperplane boundary support vector.

Finally, find the point closest to each support vector, and judge if the closest distance is consistent with the classification of the vector; then, keep it, or otherwise delete it.

This article uses the Euclidean distance to measure the distance between two points. For high-dimensional data, the distance between two points is the distance of two vectors, for example, $X=(x_{1},x_{2},\cdots ,x_{n})$ and $Y=(y_{1},y_{2},\cdots ,y_{n})$, *X* and *Y* distance D(X,Y) is written as:

(18) $$D\left(X,Y\right)=\sqrt{\sum_{i=1}^{n}{\left(x_{i},y_{i}\right)}^{2}},1\leq i\leq n.$$

Step 3: Reorganizing processing data, and obtaining new data.  

SVM is mainly used for two types of data. This article mainly uses multiple categories of data. First of all, the data in multiple categories were put into pairs, respectively. Then, two kinds of data are pruned with SSVM. The data processing is divided into the following steps:

(1) Construct hyperplane for training.
(2) Test the data with a trained hyperplane.
(3) Set the appropriate threshold to find out the classification of the training data and training results below the threshold.
(4) Finding the nearest neighbor of each support vector form data obtained in step (3), calculating the distance between the support vectors and the data points, and setting the distance between the points to itself be infinity.
(5) Find the nearest vector point of each support vector.
(6) Determine whether the support vector is consistent with its corresponding nearest neighbor vector classification result, and mark it as 0 if inconsistent.
(7) Remove the data marked as 0 in the data.
(8) Reorganize data to get new data.

According to the description of the SSVM, the SSVM pruning algorithm is the most important part of the SSVM. The details of SSVM pruning algorithm are shown in Algorithm 1.

**Algorithm 1** SSVM pruning algorithm.**Input:**  The selected training sample 〈X,Y〉, $X=(X_{1},X_{2},\cdots ,X_{n})$;**Output:**  Trimmed sample 〈X1,Y1〉  1:      **Begin**  2:     Obtain support vector 〈Z,H〉 by SVM, $Z=(Z_{1},Z_{2},\cdots ,Z_{n})$  3:         **for** i:=1 to n **do**  4:             **for** i:=1 to m **do**  5:                   Calculate the distance $D(Z_{i},X_{j})$ between Z and X by Equation (18), When $Z_{i}$ is the same as $X_{j}$, define the distance D as infinite.  6:             **end**  7:              Find the nearest dimension vector $X_{j}$ between $Z_{i}$ and *X*  8:              Judge whether $H_{i}$ and $Y_{j}$ are the same, if not, let $Y_{j}$ = 0  9:          **end**  10:          Delete the sample data of the Y=0  11:         **return**
〈X1,X2〉  12:    **end**

## 4. Experiment and Analysis

### 4.1. Bearing Data Preprocessing

**Figure 5:**
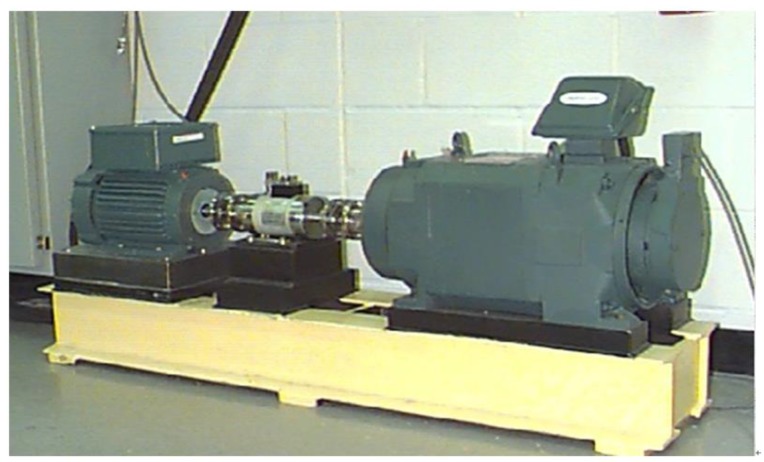
Experimental diagram of experimental platform for rolling bearing fault.

The data in this article is from bearing fault signals provided by the Case Western Reserve University (CWRU) laboratories [34]. The experimental platform is shown in Figure 5. The experimental platform consists of a torque tachometer, a 1.5 KW motor and a dynamometer. The experimental data uses the acceleration signals collected by the acceleration sensors. The sensor is fixed to the position of the driving end and the fan end of the motor shell at 12 o’clock with the magnetic base, and the vibration signal is collected through the recorder. The type of bearing used in the test is SKF6205-2RS deep groove ball bearing. The sampling frequency of the experiment is 12 KHz, the speed is 1797 rpm, and the main data is collected from normal vibration signal and the fault vibration signal.

**Figure 6:**
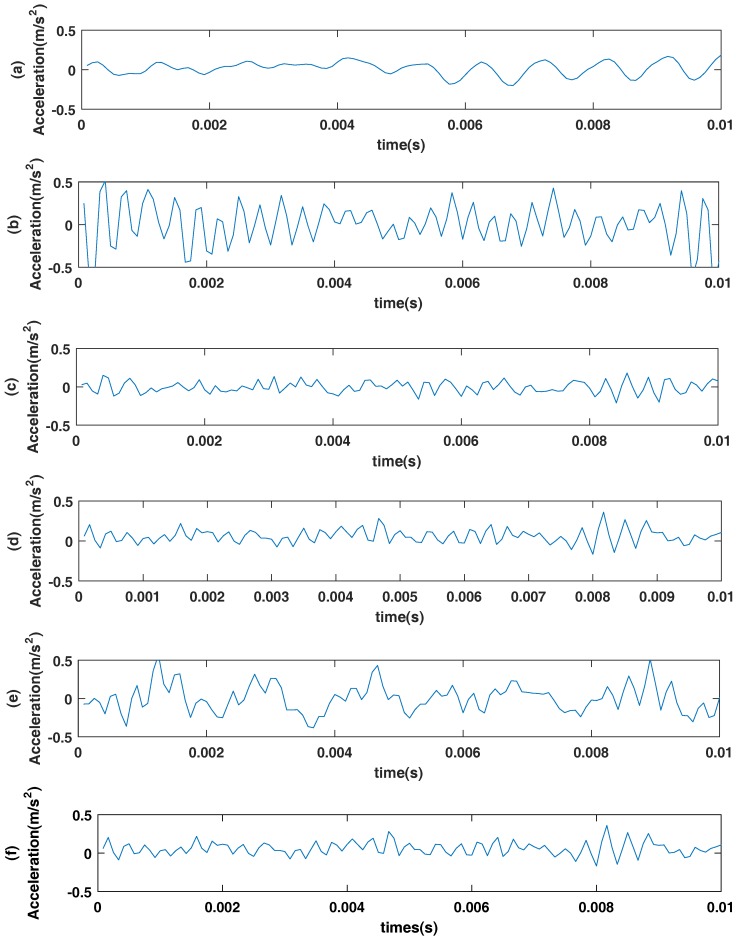
The time domain waveform of rolling bearings is shown in the figure. The *x*-axis is the time unit of the second and *y*-axis is the driving end bearing accelerator data. (**a**) normal bearing signal waveform; (**b**) inner fault signal waveform; (**c**) roller fault signal waveform; (**d**) outer fault signal waveform at center @6:00; (**e**) outer ring fault signal at orthogonal @3:00; (**f**) outer fault signal waveform at opposite @12:00.

In this paper, the normal vibration signals and fault signals of bearings are analyzed, and the samples of each type of signals are at least 12,100. The main samples of this paper are those samples with no load and the 0.021 (inches) radius fault. Table 1 describes a normal bearing signal and five kinds of fault bearing signals used in this paper. Six kinds of bearing data are described in Figure 6.

### 4.2. Application of Improved Algorithm in Bearing Fault

Fault diagnosis model is constructed according to Figure 2. This paper chooses West University of rolling bearing samples and the numbers of each state are at least 121,200. Test data and training data account for half of the total data. The detailed description of various bearing States is shown in Table 2.

In this paper, the vibration signal is mainly processed from three aspects. 

First, the feature extraction is performed by the time domain method. 

The statistical characteristics of signal vibration amplitude will change with the location and the size of the fault. The time domain waveform is dynamically transformed over time. The amplitude of the vibration signal can reflect the characteristic information of the signal intuitively. The time domain waveform information can be used to diagnose the state of the bearing by analyzing the amplitude, shape and other characteristics of the waveform. The time domain characteristic parameters are different due to different fault types and different fault degree. Generally speaking, the time domain feature provides the global characteristics of bearing state, and can effectively extract the bearing fault feature.

In the actual situation, there is various information of bearing fault, and a faults are often accompanied with other faults, such as bearing deformation, corrosion and so on. In order to diagnose the fault more effectively, we need to extract the feature of bearing fault data. In this paper, 17-time domain extraction methods are used to extract the features of the signal.

In Table 3, X(n) is the representative of the signal sample n=1,2,…,m, and *m* represents the number of samples. Seventeen time domain feature attributes is: $T_{1}$ the average value, $T_{2}$ absolute mean, $T_{3}$ effective value, $T_{4}$ average power, $T_{5}$ square amplitude, $T_{6}$ peak, $T_{7}$ peak-to-peak, $T_{8}$ variance, $T_{9}$ standard deviation, $T_{10}$ skewness, $T_{11}$ kurtosis, $T_{12}$ waveform, $T_{13}$ Crest index, $T_{14}$ impluse index, $T_{15}$ margin index, $T_{16}$ skewness index and $T_{17}$ kurtosis index. 

Second, the main feature selection of feature extraction data is made by the Decision Tree. 

**Figure 7:**
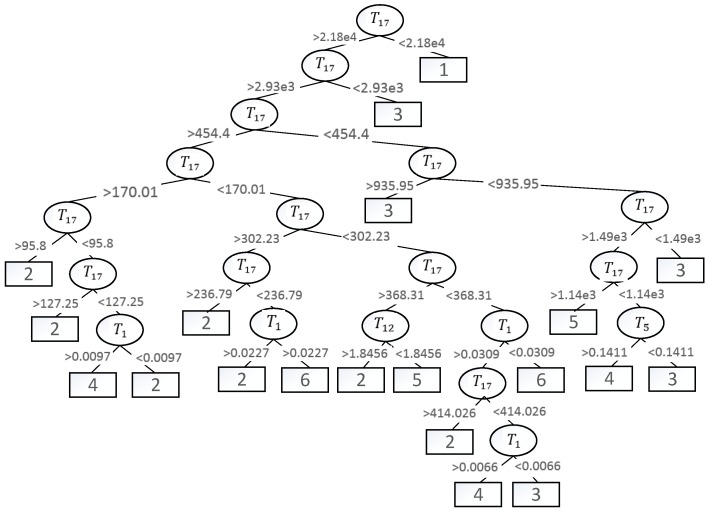
A part of the Decision Tree.

The main description of the J48 algorithm is given in Chapter 3, and the output tree structure shown in Figure 7. It can be seen from the diagram that the main characteristics of bearing data are $T_{1}$, $T_{5}$, $T_{12}$ and $T_{17}$.

The 17 characteristic attributes obtained by feature extraction are interrelated with each other, which leads to data redundancy. The attributes with low correlation are obtained by extracting the main features with J48 so that the independence of data can be enhanced.

The description and significance of these four main time-domain features are as follows:

- average value $\left(T_{1}\right)$: $T_{1}$ is mainly used to reflect the trend of the bearing fault signal,
- square amplitude $\left(T_{5}\right)$: $T_{5}$ is mainly used to describe the energy of signals,
- waveform index $\left(T_{12}\right)$: $T_{12}$ is sensitive to fault signals with stable waveform,
- kurtosis index $T_{17}$: kurtosis is sensitive to bearing defects and can reliably reflect the state of rolling bearings. It is not easy to be affected by temperature, speed, etc. and comprehensive analysis of kurtosis, peak factor, and effective value.

In Figure 7, the intermediate node represents the attribute of the decision with an ellipse, and the leaf node represents the classification result with a rectangle. The data between nodes are the classification condition. The graph is a part of the Decision Tree. Class label is a class with the highest probability in classification result when it has little effect on feature selection. 

Third, the main feature of extraction is pruned with SSVM. 

The J48 algorithm is mainly used to extract attribute vector so that the connection between data is reduced and the independence between data is enhanced. This paper mainly uses SSVM as mentioned above to reduce the similar attributes on the data dimension. The more similar the attribute is, the more redundant it would be. The data redundancy between the pruned data will be reduced so that the independence of the data dimension can be enhanced.

**Figure 8:**
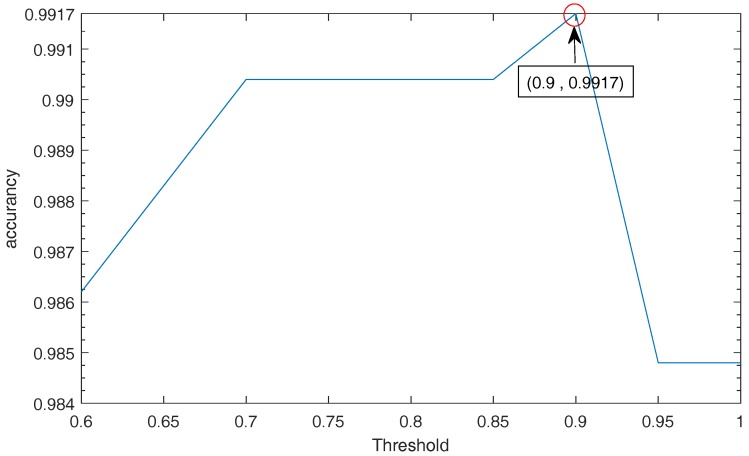
The accuracy of the data corresponding to the threshold.

SSVM is used to select the appropriate data for pruning. When the data is removed excessively or removed too little, the classification result will be affected. Therefore, it is very important to choose the appropriate threshold. The threshold in this article is the accuracy rate of test data tested by SVM. When the accuracy is greater than a certain value, we think that these kinds of data are not redundant, so we do not prune it. Therefore, the classification data, which is below the threshold, is selected, and then remove the nearest neighbor inconsistent data. Table 4 shows the selected data corresponding to the pruning data and the pruned training data set, and Figure 8 is the test accuracy of the bearing data corresponding to the selection threshold. From Table 4 and Figure 8, it can be concluded that the data trimming is too small to make the classification effect not obvious, and too much data pruning will result in important data loss. It can be seen from Figure 8 that, when the threshold is 0.9, the corresponding accuracy is the highest than others. Therefore, the training data with a threshold below 0.9 is selected for SSVM pruning. Only in this way can the fault diagnosis be performed effectively.

**Figure 9:**
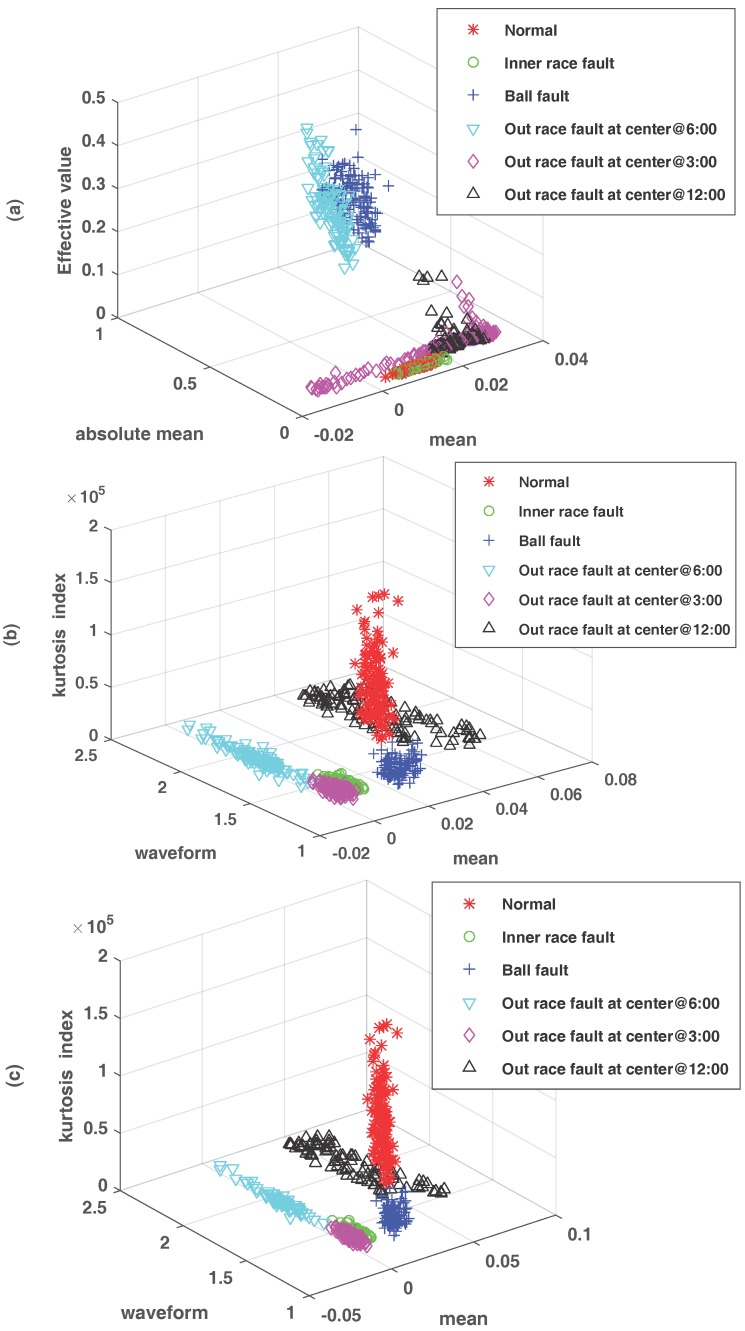
Bearing data description. (**a**) the original signal time domain feature extraction fault three-dimensional; (**b**) J48 select the characteristics of the three-dimensional fault data diagram; (**c**) the three-dimensional fault data diagram after J48 and SSVM pruning.

After processing, the vibration data from the three aspects above, the redundant data is removed from the feature vector and the dimension vector, respectively. Figure 9 shows the three-dimensional data of time domain feature extractiont, three-dimensional data after J48 select feature, and three-dimensional data after J48 and SSVM trimming. The axes *x*, *y*, and *z* in Figure 9 are dimensional features. Among them, Figure 9a selects three dimensions of mean, absolute mean and effective value, Figure 9b,c select three dimensions of mean, waveform index and kurtosis index. It can be seen from Figure 9 that each class of data has obvious overlap in Figure 9a, the overlap ratio of each kind of data in Figure 9b is obviously lower than of Figure 9a, and Figure 9c obviously separates each type of category data. Therefore, it is shown from Figure 9 that the redundancy between the processed data is greatly reduced, so that the correlation between the data is reduced, and the influence of NB independence assumption on the fault diagnosis is finally reduced.

The processing bearing fault data correlation is low, which reduces the limitation of the independence assumption on NB fault diagnosis. Table 5 is the confusion matrix of NB fault diagnosis for the processed data, and Table 6 is a confusion matrix for bearing fault diagnosis using an NB model without redundant vibration data. As can be seen from the table, the model has been improved for each category after redundancy removal.

**Figure 10:**
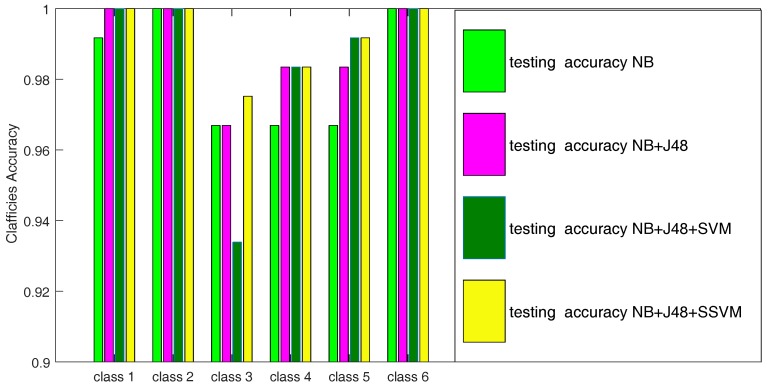
Testing accuracy comparison of each condition in the experiment.

In order to verify the validity of this algorithm in bearing data, the data simulation is carried out by MATLAB (Version 8.6, The MathWorks, MA, USA). Figure 10 and Table 7 are bearing fault diagnosis results. In Figure 10 and Table 7,the meaning of NB+J48+SVM is that first data is selected by J48,then the data after feature selection is pruned by SVM and the fault diagnosis of NB is finally carried out. Compared with other experimental results, the bearing fault diagnosis experimental results on JSSVM-NB is better than removing the data redundancy by feature vector and data vector. Compared with other experiments, the accuracy of the fault diagnosis model is 99.17%. Table 8 shows the comparison of results of about JSSVM-NB and reference [35], which have the same data for bearing fault diagnosis. It can be seen from Table 7 and Table 8 that the JSSVM-NB model is effective for rolling bearing fault diagnosis.

## 5. Conclusions

In this paper, in order to improve the independence assumption, the bearing data processing is carried out from two aspects of the attribute vector and the dimension vector, and the bearing data with higher data independence is obtained for the bearing fault diagnosis of the NB. NB is based on the conditional independence hypothesis of Bayes. However, in the actual case, it is difficult for the bearing data vector to achieve independence. Therefore, the redundancy is removed from the feature attribute vector and dimension of bearing data in this paper, so that the connection between data is reduced and the bearing condition monitoring on NB be enhanced. It be seen from the simulation results. The NB improved the data independence has realized the fault diagnosis of the different parts of rolling bearing, and can be applied to the other fault diagnosis of the industrial.

## Figures and Tables

**Table 1:** Description of CWRU dataset.

Data Type	Motor Load (HP)	Fault Diameter (Inches)	Label
Normal	0	0	1
Inner race	0	0.021	2
Ball	0	0.021	3
Out race fault at center @6:00	0	0.021	4
Out race fault at orthogonal @3:00	0	0.021	5
Out race fault at opposite @12:00	0	0.021	6

**Table 2:** Description of the data sets.

Data Type	The Number of Training	The Number of Testing	Label
Normal	121	121	1
Inner race	121	121	2
Ball	121	121	3
Out race fault at center @6:00	121	121	4
Out race fault at orthogonal @3:00	121	121	5
Out race fault at opposite @12:00	121	121	6

**Table 3:** Time domain analysis of bearing fault data.

Number	Characteristic Equation	Number	Characteristic Equation
1	$T_{1}=\frac{\sum_{n=1}^{d}X\left(n\right)}{d}$	2	$T_{2}=\frac{\sum_{n=1}^{d}\left|X\left(n\right)\right|}{d}$
3	$T_{3}=\sqrt{\frac{\sum_{n=1}^{d}{\left(X\left(n\right)\right)}^{2}}{d}}$	4	$T_{4}=\frac{\sum_{n=1}^{d}{\left(X\left(n\right)\right)}^{2}}{d}$
5	$T_{5}={\left(\frac{\sum_{n=1}^{d}\sqrt{\left|X\left(n\right)\right|}}{d}\right)}^{2}$	6	$T_{6}=max\left|X(n)\right|$
7	$T_{7}=max\left(X\left(n\right)\right)-min\left(X\left(n\right)\right)$	8	$T_{8}=\frac{\sum_{n=1}^{d}{\left(X\left(n\right)-T_{1}\right)}^{2}}{d-1}$
9	$T_{9}=\sqrt{\frac{\sum_{n=1}^{d}{\left(X\left(n\right)-T_{1}\right)}^{2}}{d-1}}$	10	$T_{10}=\frac{\sum_{n=1}^{d}{\left(X\left(n\right)\right)}^{3}}{d}$
11	$T_{11}=\frac{\sum_{n=1}^{d}{\left(X\left(n\right)\right)}^{4}}{d}$	12	$T_{12}=\frac{T_{3}}{T_{2}}$
13	$T_{13}=\frac{T_{6}}{T_{3}}$	14	$T_{14}=\frac{T_{6}}{T_{2}}$
15	$T_{15}=\frac{T_{6}}{T_{5}}$	16	$T_{16}=\frac{T_{10}}{T_{9}^{3}}$
17	$T_{17}=\frac{T_{11}}{T_{9}^{4}}$		

**Table 4:** The corresponding threshold data.

Threshold (Training Accuracy)	1	0.95	0.90	0.85	0.80	0.60
**The number of pruning**	190	187	179	155	102	0
**The number of training**	536	539	547	571	624	726

**Table 5:** Confusion matrix of the processing bearing fault data on test sets.

Actual Classes	Predicted Classes					
	1	2	3	4	5	6
1	121	0	0	0	0	0
2	0	121	0	0	0	0
3	0	0	121	0	0	0
4	0	0	0	121	0	0
5	0	0	0	0	121	0
6	0	0	0	0	0	121

**Table 6:** Confusion matrix of NB on test sets.

Actual Classes	Predicted Classes					
	1	2	3	4	5	6
1	120	0	1	0	0	0
2	0	121	0	0	0	0
3	0	0	117	0	0	4
4	0	1	0	117	2	1
5	0	1	0	3	117	0
6	0	0	0	0	0	121

**Table 7:** The corresponding threshold data.

Methods	Accuracies
**NB**	98.21%
**NB + J48 + SVM**	98.48%
**NB + J48 + SSVM (JSSVM-NB)**	99.17%

**Table 8:** The comparison results in bearing fault diagnosis.

State	JSSVM-NB	Reference [35]
Normal	100%	98.31%
Inner race	100%	97.73%
Ball	97.5%	95.04%
Out race fault at center (@6:00, @3:00 and @12:00)	99.17%	98.02%
